# Hydroxychloroquine‐induced repetitive atypical pustular drug eruptions in the same patient separated by 12 years

**DOI:** 10.1002/ccr3.7677

**Published:** 2023-07-21

**Authors:** Jeffrey Chen, Lauryn M. Falcone, Arivarasan D. Karunamurthy, Jonhan Ho, Joseph C. English

**Affiliations:** ^1^ University of Pittsburgh School of Medicine Pittsburgh Pennsylvania USA; ^2^ Department of Dermatology University of Pittsburgh Pittsburgh Pennsylvania USA; ^3^ Dermatopathology Section Unviversity of Pittsburgh Pittsburgh Pennsylvania USA

**Keywords:** acute generalized exanthematous pustulosis, drug eruption, hydroxychloroquine, pustular psoriasis, pustulosis

## Abstract

Hydroxychloroquine (HCQ) has been reported to cause pustular drug eruptions such as acute generalized exanthematous pustulosis (AGEP) and putular psoriasis (PP). Clinical differentitation of these entities is often difficult. This case emphasizes characteritics of AGEP and PP as well as the need for clinicans to proactively follow‐up these patients to monitor for the more aggressive outcome of PP.

## Graphical abstract

Pustular drug eruption from hydroxychloroquine (HCQ) initial presentation (Figure 1 A/B) recurring 12 years later after HCQ was re‐introduced (Fig 1 C/D) for underlying connective tissue disease.

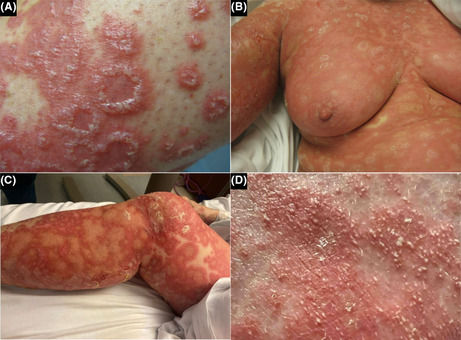

## 1 INTRODUCTION

Acute generalized exanthematous pustulosis (AGEP) and pustular psoriasis (PP) are severe skin conditions with overlapping features making them difficult to distinguish. AGEP is usually drug‐related and characterized by an acute generalized eruption of non‐follicular sterile micropustules on an erythematous base, fever, and neutrophilia.^1^ PP can be idiopathic, pregnancy‐ or drug‐related and presents as erythema with confluent pustules that lead to desquamation and laboratory abnormalities.^2^ Drugs purported to be causative agents in pustular reactions are primarily based on case reports in drug‐naive patients, making it difficult to discriminate drug hypersensitivity reactions, like AGEP, from de novo drug‐induced PP. Herein, we report an unusual case of hydroxychloroquine (HCQ)‐induced atypical pustular drug eruption in a patient that had a similar reaction to HCQ 12 years prior.

## 2 CASE REPORT

**FIGURE 1:**
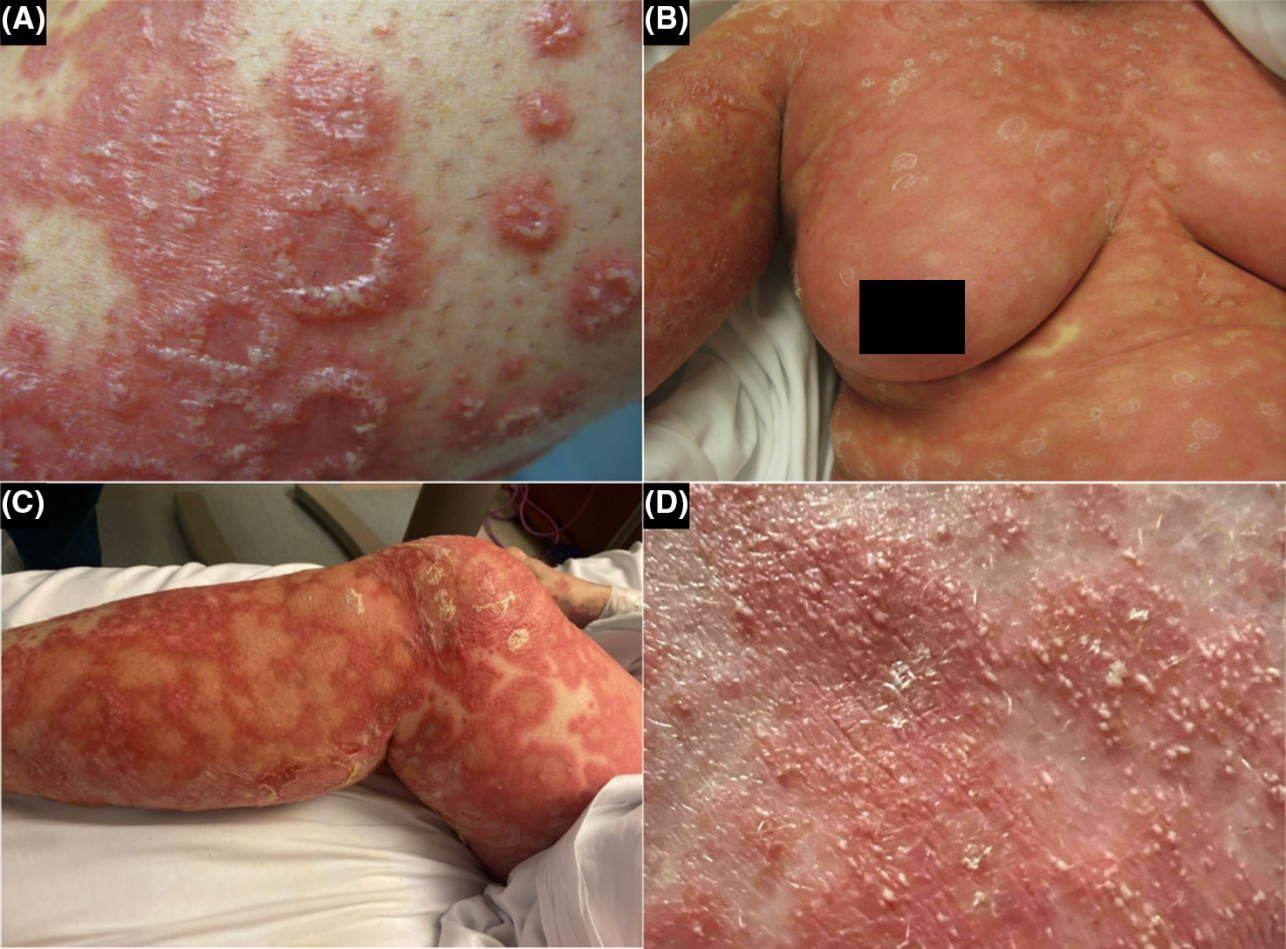
(A) 2010 extremity, (B) 2010 torso, (C) 2022 extremity, (D) 2022 torso.

A 70‐year‐old female patient with a history of undifferentiated connective tissue disorder and rheumatoid arthritis (RA) presented to dermatology in 2010 with a severe generalized bullous and pustular rash (Figure 1). The rash developed after initiation of HCQ for her RA which was immediately discontinued. Biopsy of the right forearm revealed a large subepidermal blister with neutrophils, eosinophils, and several subcorneal pustules. Due to suspected drug exanthem secondary to HCQ, the patient was switched to mycophenolic acid for her RA and was prescribed prednisone for her rash. The pustules significantly improved within 3 months.

**FIGURE 2:**
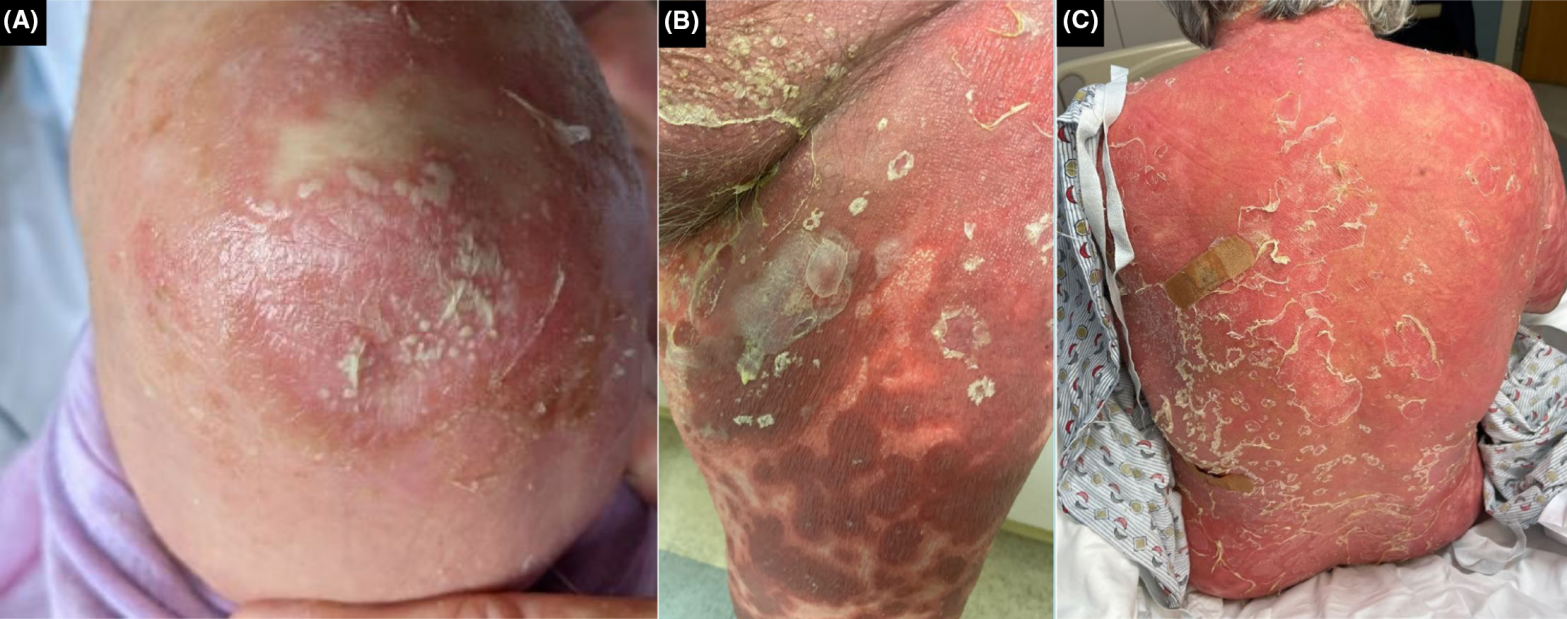
Diffuse erythema, desquamation, and progressive pustulation in 2022 of the (A) elbow, (B) left leg, (C) back.

**FIGURE 3:**
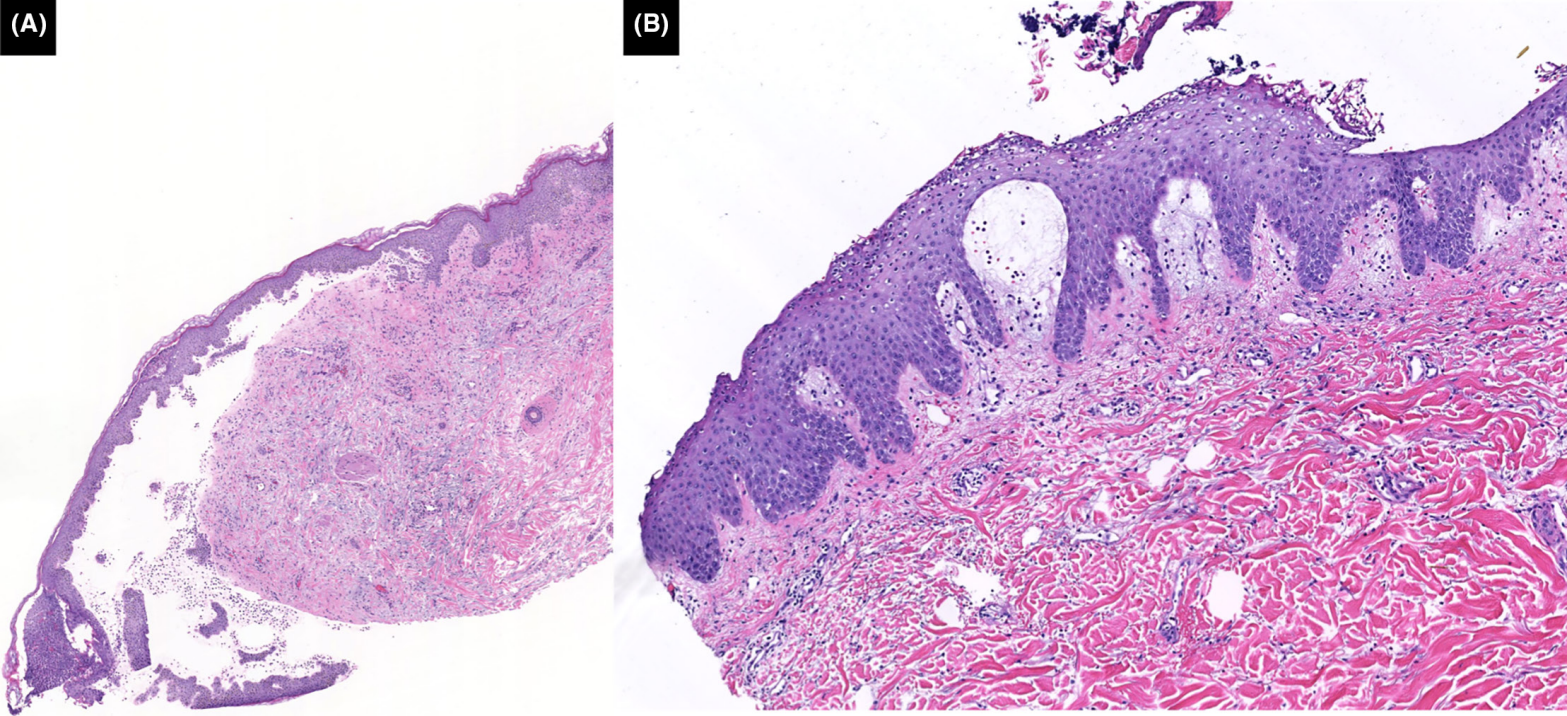
(A) Subepidermal blister with neutrophils and eosinophils, and several subcorneal pustules from 2010; H&E stain at 2×. (B) Subcorneal collections of neutrophils and underlying spongiotic epidermis with papillary dermal edema and superficial perivascular and interstitial infiltrate composed of lymphocytes, histiocytes, neutrophils, and rare eosinophils from 2022; H&E stain at 10×.

Unsure if her eruption in 2010 was truly a drug reaction, rheumatology restarted her on HCQ in 2022 due to persistent difficult‐to‐treat RA. Three days after starting HCQ, the patient was admitted to the hospital for widespread erythema and pustulation (Figures 1 and 2). On admission, she denied any history of psoriasis. Abnormal laboratory values reflected marked leukocytosis, hyperglycemia, hypoproteinemia, transaminitis, and elevated C‐reactive protein. Biopsies taken from the back showed similar features and revealed separation of the stratum corneum with multifocal conspicuous subcorneal collections of neutrophils and underlying spongiotic epidermis with neutrophilic exocytosis. Papillary dermal edema and superficial perivascular and interstitial infiltrate composed of lymphocytes, histiocytes, neutrophils, and rare eosinophils were observed (Figure 3).

The differential diagnosis included AGEP and PP, but initial clinicopathologic features were most consistent with AGEP. Accordingly, the patient was given intravenous methylprednisolone and was prescribed prednisone, triamcinolone acetonide cream, and cyclosporine. She continued to experience recurrent flares over the next 2 months with pustules coalescing into irregular lakes of pus. Due to the worsening flares, the patient was eventually started on ixekizumab, and her rash completely resolved within 2 months. Given the history of prior pustular eruption in the setting of HCQ use, expanding pustulation, and rash persistence, a diagnosis of HCQ‐induced pustular drug eruption favoring PP over AGEP was concluded.

## 3 DISCUSSION

Antimalarial drugs have been associated with AGEP and PP.^3^, ^4^, ^5^, ^6^, ^7^ Laboratory findings such as leukocytosis, hypoalbuminemia, electrolyte abnormalities, elevated C‐reactive protein, and elevated liver enzymes may be present in both conditions.^1^, ^2^, ^8^, ^9^ Histopathologically, eosinophilic spongiosis, dermal eosinophilia, papillary dermal edema, and a mixed interstitial and perivascular infiltrate may favor AGEP over PP, although a diagnosis is often made clinically.^1^, ^10^ A negative history of psoriasis, previous drug reaction, and/or shorter duration of pustules (<15 days) prefer AGEP over PP. Given these factors, our patient's presentation was initially suggestive of AGEP (known culprit drug: HCQ, neutrophilia, negative history of psoriasis, preceding drug reaction, and histological findings).^1^ The rapid relapse, duration, and morphological changes of her rash, however, corroborated the diagnosis of HCQ‐induced PP. While AGEP is often self‐limiting, PP is associated with high morbidity and can be life‐threatening without rapid treatment.^1^, ^2^, ^9^ This case highlights the need to reappraise differential diagnoses of AGEP vs PP within their appropriate and evolving clinical contexts due to the therapeutic and prognostic implications.

## AUTHOR CONTRIBUTIONS

**Jeffrey Chen:** Conceptualization; investigation; methodology; project administration; validation; visualization; writing – original draft; writing – review and editing. **Lauryn M. Falcone:** Supervision; writing – review and editing. **Arivarasan D. Karunamurthy:** Resources; writing – review and editing. **Jonhan Ho:** Resources; writing – review and editing. **Joseph C. English:** Conceptualization; supervision; writing – review and editing.

## FUNDING INFORMATION

None.

## CONFLICT OF INTEREST STATEMENT

All authors report no conflict of interest concerning the materials or methods used in this study or the findings specified in this paper. This research did not receive any specific grant from funding agencies in the public, commercial, or not‐for‐profit sectors.

## CONSENT

Written informed consent was obtained from the patient to publish this report in accordance with the journal's patient consent policy.

## Data Availability

Data sharing is not applicable to this article as no datasets were generated or analyzed during the current study.
